# Lactate Increases Renal Cell Carcinoma Aggressiveness through Sirtuin 1-Dependent Epithelial Mesenchymal Transition Axis Regulation

**DOI:** 10.3390/cells9041053

**Published:** 2020-04-23

**Authors:** Vera Miranda-Gonçalves, Ana Lameirinhas, Catarina Macedo-Silva, João Lobo, Paula C. Dias, Verónica Ferreira, Rui Henrique, Carmen Jerónimo

**Affiliations:** 1 Cancer Biology & Epigenetics Group—Research Center, Portuguese Oncology Institute of Porto (CI-IPOP), 4200-072 Porto, Portugal; vera.miranda.goncalves@ipoporto.min-saude.pt (V.M.-G.); ana.lameirinhas@ipoporto.min-saude.pt (A.L.); ana.catarina.macedo.silva@ipoporto.min-saude.pt (C.M.-S.); jpedro.lobo@ipoporto.min-saude.pt (J.L.); paula.dias@ipoporto.min-saude.pt (P.C.D.); veronicaf@ipoporto.min-saude.pt (V.F.); henrique@ipoporto.min-saude.pt (R.H.); 2 Master in Oncology, Institute of Biomedical Sciences Abel Salazar, University of Porto (ICBAS-UP), 4050-313 Porto, Portugal; 3 Department of Pathology, Portuguese Oncology Institute of Porto, 4200-072 Porto, Portugal; 4 Department of Pathology and Molecular Immunology, Institute of Biomedical Sciences Abel Salazar–University of Porto (ICBAS-UP), 4050-313 Porto, Portugal

**Keywords:** renal cell carcinoma, Warburg effect, lactate, epigenetic regulation, sirtuin 1

## Abstract

Background: Renal cell carcinoma (RCC) displays a glycolytic phenotype (Warburg effect). Increased lactate production, impacting on tumor biology and microenvironment modulation, has been implicated in epigenetic mechanisms’ regulation, leading to histone deacetylases inhibition. Thus, in-depth knowledge of lactate’s impact on epigenome regulation of highly glycolytic tumors might allow for new therapeutic strategies. Herein, we investigated how extracellular lactate affected sirtuin 1 activity, a class III histone deacetylase (sirtuins, SIRTs) in RCC. Methods: In vitro and in vivo interactions between lactate and SIRT1 in RCC were investigated in normal kidney and RCC cell lines. Finally, SIRT1 and N-cadherin immunoexpression was assessed in human RCC and normal renal tissues. Results: Lactate inhibited SIRT1 expression in normal kidney and RCC cells, increasing global H3 and H3K9 acetylation. Cells exposed to lactate showed increased cell migration and invasion entailing a mesenchymal phenotype. Treatment with a SIRT1 inhibitor, nicotinamide (NAM), paralleled lactate effects, promoting cell aggressiveness. In contrast, alpha-cyano-4-hydroxycinnamate (CHC), a lactate transporter inhibitor, reversed them by blocking lactate transport. In vivo (chick chorioallantoic membrane (CAM) assay), lactate and NAM exposure were associated with increased tumor size and blood vessel recruitment, whereas CHC displayed the opposite effect. Moreover, primary RCC revealed N-cadherin upregulation whereas SIRT1 expression levels were downregulated compared to normal tissues. Conclusions: In RCC, lactate enhanced aggressiveness and modulated normal kidney cell phenotype, in part through downregulation of SIRT1, unveiling tumor metabolism as a promising therapeutic target.

## 1. Introduction

Renal cell carcinomas (RCC), specifically clear cell RCC (ccRCC), are the most lethal among common urological tumors. Although most RCCs are currently diagnosed at early stages, about 20% of patients already harbor metastases at diagnosis, decreasing 5 year survival rates to less than 10% [1,2,3]. Despite therapeutic advances, the median survival of these patients remains low. Thus, improving metastatic RCC patients’ prognosis remains a challenge.

Metabolic reprogramming, a cancer hallmark, plays a critical role in neoplastic development and progression [4]. Tumor cells undergo a metabolic switch to aerobic glycolysis (Warburg effect) leading to increased glucose consumption [5]. In fact, glycolysis supports several biosynthetic pathways required for normal cell division and tumor proliferation [6]. RCC cells are highly dependent of glycolytic metabolism and consequently produce large amounts of lactate, resulting in its accumulation in the tumor microenvironment [7]. Several studies implicated lactate production in tumor progression and therapy resistance, also contributing to angiogenesis, metastization, and immune escape, as well as radioresistance [8,9]. Moreover, a tumor metabolic symbiosis hypothesis [10] has been proposed, claiming that lactate might be used as an energy source in the tumor microenvironment [11].

Epigenetic deregulation, due to changes in DNA methylation, histone post-translational modifications, and chromatin remodeling complexes, is a hallmark in several human diseases [12,13], including cancer [14]. Recently, cell metabolism was shown to regulate epigenetic machinery, as metabolites are able to modulate epigenetic enzymes involved in histone post-translational modifications, including acetylation and methylation [15,16]. Specifically, inhibition of histone deacetylase (HDAC) activity by lactate was reported in colon and cervical cancers [17,18]. HDACs are categorized into four classes. Whereas classes I, II, and IV are all zinc-dependent enzymes that catalyze lysine-amino bond hydrolysis [19], HDAC class III (sirtuins (SIRTs)) deacetylation activity relies on NAD^+^ [20]. Hence, HDACs are responsible for loss of acetylation on N-terminal tails of histone H3 and H4, leading to chromatin condensation by restoring the positive charges of lysine with consequent transcription silencing [21], increasing tumorigenesis and tumor aggressiveness [22]. Additionally, SIRTs modulate several other cellular processes, including cellular metabolism, genomic stability maintenance, cell proliferation, and epithelial–mesenchymal transition (EMT) phenotype [23,24]. Nuclear SIRT1, the most studied member of the SIRT family, is variably considered an oncogene or tumor suppressor gene depending on tumor context [25,26]. Concomitantly, SIRT1 is associated with EMT regulation, being involved in cancer progression and metastasis [27]. In RCC, the involvement of SIRT1 in aggressive phenotype modulation has not been described yet. Thus, we sought to uncover the effect of lactate in epigenetic modulation of RCC aggressiveness, as well as the ability to epigenetically modulate adjacent normal cells. For that purpose, lactate effects upon epigenetic enzymes, particularly SIRT1, and consequent alterations in tumor cell aggressiveness were firstly evaluated. Then, normal kidney cells were exposed to lactate and conditioned medium (CM) from tumor cells to assess the putative effect of lactate in neoplastic transformation. Furthermore, specific epigenetic enzymes and monocarboxylate transporter (MCT) inhibitors were used to further support lactate’s role in epigenetic deregulation. Finally, these findings were assessed in vivo, using the chick chorioallantoic membrane (CAM) assay and validated in a series of primary RCC and normal kidney tissues.

## 2. Materials and Methods

### 2.1. Cell Lines Culture

Epithelial human kidney cell lines (HKC-8, 786-O, Caki-1, Caki-2, and ACHN) were obtained from American Type Culture Collection (Table S1). To normalize glucose quantity available, all cell lines were maintained in RPMI-1640 medium (Biochrom, MERK, Darmstadt, Germany) supplemented with 10% fetal bovine serum (Biochrom, MERK, Germany) and 1% penicillin/streptomycin (GBICO, Invitrogen, Carlsbad, CA, USA) at 37 °C and 5% CO_2_ in a humidified chamber. Mycoplasma test was performed using TaKaRa PCR Mycoplasma Detection Set (Clontech Laboratories, EUA, Brussels, Belgium), before all experiments.

### 2.2. Tissue Samples

A series of 80 RCC tissue samples comprising ccRCC and papillary RCC (pRCC) subtypes (40 cases each), collected from patients submitted to radical or partial nephrectomy at the Portuguese Oncology Institute of Porto, Portugal, between 2003 and 2007, were included in this study. Additionally, a set of 30 morphologically normal kidney (cortical) samples from nephrectomy specimens obtained from patients with low stage upper urinary tract urothelial carcinoma were used as controls. This study was approved by the Institutional Review Board (Comissão de Ética para a Saúde) of the Portuguese Oncology Institute of Porto (CES IPO: 372/2017).

### 2.3. Chemicals

Sodium l-lactate (LAC, L7022-5G; Sigma-Aldrich, Darmstadt, Germany) was dissolved in sterile distilled water (B. Braun, Melsungen, Germany) to a 1 M stock solution and stored at 4 °C until used. Nicotinamide (NAM; Sigma-Aldrich, Darmstadt, Germany) was dissolved in sterile distilled water to 400 mM stock solution and stored at 4 °C until further use. From this, a 20 mM intermediate stock solution was obtained. Alpha-cyano-4-hydroxycinnamate (CHC; Sigma-Aldrich, Germany) was dissolved in dimethyl sulfoxide (DMSO; Sigma-Aldrich, Darmstadt, Germany) to a 3 M stock solution, from which intermediate stock solutions (0.1–1.5 M) were prepared and stored at −20 °C until used. Working solutions were freshly prepared in complete RPMI-1640 medium. For working solutions of CHC, DMSO concentration did not exceed 1%. Control condition for CHC was performed with 1% DMSO in complete RPMI-1640 medium.

### 2.4. Lactate Supplementation

Cells were plated in 25 cm^3^ culture flasks in complete RPMI-1640 medium at an optimal density in order to obtain an 80–90% confluence at the ending timepoint. After cells’ exposure to 20 mM lactate in complete RPMI-1640 medium for 48 h, RNA and protein were extracted using standard methods.

### 2.5. Conditioned Medium

Complete RPMI-1640 medium was used to culture 786-O Caki-1, Caki-2, and ACHN cells for 72h. After this period, CM was collected, centrifuged, and stored at −80 °C until use. The same procedure was performed after RCC cell lines’ treatment with CHC, as described in Section 2.7. Then, lactate and glucose from each CM were quantified.

Two human renal proximal tubule epithelial cell lines (HKC-8 and HK-2) were seeded in 25 cm^3^ culture flasks in complete RPMI-1640 medium at the same density used for lactate exposure. Then, culture medium was replaced by a combination of 72 hours CM from tumor cell lines with complete Eagle’s minimum essential medium (Eagle’s MEM; Biochrom, MERK, Darmstadt, Germany), in a 2:1 ratio, using Eagle’s MEM as control condition. After 48 h, RNA and protein extraction were performed.

### 2.6. SIRT Inhibition

The five kidney cell lines were plated in 25 cm^3^ culture flasks in complete RPMI-1640 medium and, after cell adhesion, 200 µM NAM was added to promote SIRT inhibition, and RNA and protein extraction was then performed 24 h later.

### 2.7. MCT Inhibition

CHC IC_50_ value (drug concentration that corresponds to 50% of cell viability/growth inhibition) was determined for each cell line by plating the cells at a density of 2 × 10^4^ cell/mL in 96-well plates, in complete RPMI-1640 medium and incubated at 37 °C, 5% CO_2_. Then, cells were treated with a 1-15 mM range of CHC concentrations in complete RPMI-1640 medium for 48 h. The respective controls were performed with the corresponding 1% DMSO. The effect of CHC on cell growth was evaluated by 3-(4,5-dimethylthiazol-2-yl)-2,5-diphenultetrazolium (MTT) assay (Sigma-Aldrich, Darmstadt, Germany).

Cells were plated in 25 cm^3^ culture flasks and allowed to adhere overnight in complete RPMI-1640 medium. Then, cells were treated with the corresponding IC_50_/2 CHC value, or in a combination with 20 mM lactate for 48 h. RNA and protein extraction were performed.

### 2.8. Extracellular Glucose and Lactate Quantification

Extracellular glucose and lactate levels were quantified in CM using a colorimetric assay (Spinreact, Spain), according to the manufacturer’s instructions. Results are expressed as glucose grams per liter and lactate millimolar.

### 2.9. RNA Extraction, cDNA Synthesis, and RT-qPCR

RNA extraction was performed using the ribozol reagent method (TripleXtractor, GRiSP, Porto, Portugal). Then, reverse transcription was performed from 1000 ng of RNA using RevertAid RT kit (ThermoScientific Inc., Waltham, MA, USA), according to the manufacturer indications. Quantitative reverse transcription polymerase chain reaction (RT-qPCR) was performed in 384-well plates LightCycler480II (Roche Diagnostics, Risch-Rotkreuz, Suíça using 450 ng of cDNA, Xpert Fast SYBER Mastermix Blue (GE22.2501; GRiSP, Portugal), and specific primers described in Table S2. All samples were run in triplicate. Transcript levels were evaluated using the ΔΔCt method, and beta-glucuronidase [*β-GUS*), (housekeeping gene)] was used as reference gene to normalize results.

### 2.10. Western Blot and Coimmunoprecipitation

Total protein was extracted from cells using Kinexus lysis buffer (Kinexus Bioinformatics, Canada) with Triton X-100 (Sigma-Aldrich, Germany) supplemented with a protease inhibitor cocktail and sonicated in 5 cycles of 20 s ON and 20 s OFF, followed by centrifugation at 13,300 rpm for 30 min at 4 °C. Subsequently, protein concentration was determined using the Pierce BCA Protein Assay Kit (Thermo Scientific Inc., Waltham, MA, USA), according to the manufacturer’s procedures. Then, 30µg total protein was separated in 8–12.5% polyacrylamide gel by SDS-PAGE and transferred into an immunoblot nitrocellulose membrane (Bio-Rad, Hercules, CA, USA) in a 25 mM Tris-base/glycine buffer using a Trans-Blot Turbo Transfer system (Bio-Rad, USA). Membranes were blocked with 5% milk or bovine serum albumin (BSA; Santa Cruz, CA, USA) in TBS/0.1% Tween (TBS/T pH = 7.6) for 1 h at room temperature. Then, the membranes were incubated overnight at 4 °C with specific primary antibodies (Table S3). After incubation, the membranes were washed in TBS/T and incubated with secondary antibody (Bio-Rad, USA), diluted at a ratio of 1:5000, for 1 h at room temperature. The bands were visualized by chemiluminescence (Clarity WB ECL substrate, Bio-Rad, USA). Western blot result quantification using band densitometry analysis was performed using the ImageJ software (version 1.6.1, National Institutes of Health). β-actin and H3 were used for loading control. The primary antibodiy dilutions are described in Table S3.

For coimmunoprecipitations, 200 µg cell lysates of total protein were incubated with anti-acetylated-lysin antibody (9441, Cell Signaling Technology) or anti SMAD4 antibody (38454, Cell Signaling Technology) and immunoprecipitated with Protein A/G magnetic beads (16-663, Sigma Aldrich) overnight at 4 °C. The eluates were blotted with anti-SMAD4 or anti-SNAIL antibodies (Table S3).

### 2.11. Immunofluorescence

Cells were seeded in cover slips at 2500 cells per well, overnight. Then, culture medium was removed and replaced for complete culture medium (RPMI-1640 medium or Eagle’s MEM) supplemented with lactate, CM, NAM, or CHC for 48 h.

For immunofluorescence, cells were fixed 10 min in 4% paraformaldehyde (Santa Cruz, USA), following cell permeabilization with Triton X-100 0.25% in 1X phosphate-buffer saline (PBS) for 15 min. Then, cells were blocked with 5% BSA in 1× PBS, for 30 min and incubated with primary antibodies diluted in 5% BSA in 1× PBS (Table S3) overnight at room temperature. The next day, the cells were incubated with secondary antibody anti-rabbit IgG- Tetramethylrhodamine (TRITC) (T6778, Sigma-Aldrich, Germany) or anti-rabbit IgG- Fluorescein isothiocyanate (FITC) (Alexa Fluor TM 488, A11008; Invitrogene, USA), for 1h at room temperature. In the next step, cells were stained with 4′,6-diamidino-2-phenylindole (DAPI; AR1176, BOSTER Biological Technologies, Pleasanton, CA, SA in mounting medium. Pictures were taken in a fluorescence microscope Olympus IX51 with a digital camera Olympus XM10 using CellSens software (Olympus, Tokyo, Japan). Mean fluorescence intensity was assessed by ImageJ software (version 1.6.1, National Institutes of Health) and normalized for controls of each respective conditions.

### 2.12. Cell Proliferation Assay

The effect on cell proliferation was assessed by Cell Proliferation ELISA BrdU (5-bromo-2’-deoxyuridine) assay (Roche Applied Sciences, Penzberg, Germany). Two thousand cells per well were plated into 96-well plates in complete RPMI-1640 medium and incubated overnight. Then, cells were treated for 48 h, and BrdU assay was performed at 0 h and at the end of each treatment, as previously described [28].

### 2.13. Wound Healing Assay

Cells were seeded (6-well plate) in complete RPMI-1640 medium at an optimal density, and were cultured to obtain at least 95% of confluence. Then, “wounds” were made by manual scratching, and cells were washed with 1× PBS and incubated with complete cell medium (RPMI-1640 medium or Eagle’s MEM) supplemented with lactate, CM, NAM, or CHC. The wound areas were photographed at regular time points using an Olympus IX51 inverted microscope equipped with an Olympus XM10 Digital Camera System. The relative migration distance was calculated using the following formula: relative migration distance (%) = (A − B)/C × 100, in which A is the width of cell wound at 0 h incubation, B is the width of cell wound after specific hours of incubation, and C is the mean width of cell wound for 0 h of incubation. For relative migration distance, the results were analyzed using the beWound—Cell Migration Tool (Version 1.5) [28].

### 2.14. Invasion Assay

For cell invasion analysis, 15,000 cells per insert were seeded, and 24-well BD BioCoat Matrigel Invasion Chambers (BD BioSciences, San Jose, CA, USA) were used according to the published report [29].

### 2.15. Chicken Chorioallantoic Membrane (CAM) Assay

The fertilized chicken eggs (Pinto Bar, Amares, Braga Portugal) were incubated in a humidified chamber at 37 °C. On the third day of development, the egg’s air chamber was punctured, and the CAM was exposed by opening a window in the eggshell. The eggs were then incubated until the 10th day of development, in which the 786-O cells (3,000,000 cells in 25 μL of BD Matrigel Matrix Growth Factor Reduced (BD Biosciences)) were placed on the CAM. At day 15, the control group received 50 uL of complete cell medium (RPMI-1640 medium) and the LAC, NAM, and CHC groups were treated with 20 mM lactate, 200µM NAM, or 3 mM CHC, respectively, diluted in complete cell medium (RPMI-1640 medium). After 48 h, the eggs were placed at −80 °C for 10 min in order to sacrifice the chicken embryos. CAMs with tumors were dissected; fixed in 4% paraformaldehyde at room temperature; and included in paraffin for immunohistochemical (IHC) analysis of SIRT1, H3K9ac, N-cadherin (NCAD), and Ki67 expression (Table S4). To determine tumor perimeter, digital images were taken on days 15 and 17 of development in a stereomicroscope (Olympus SZX16) using a digital camera (Olympus SC180), and the in ovo tumor perimeter was measured using the CellSens software (Olympus). Before paraffin inclusion, tumors were photographed ex ovo for blood vessel count, and the blood vessel number under the tumor area was counted using ImageJ software (version 1.41; National Institute of Health).

### 2.16. Immunohistochemistry

Immunohistochemistry was performed using the Novolink Max Polymer Detection System (Leica Biosystems, Germany) or UltraVision Detection System (Large Anti-Polyvalent, HRP; Thermo Scientific Inc., USA) in 3μm thick sections from formalin-fixed and paraffin-embedded tissues. After deparaffinization and rehydration, antigen retrieval was accomplished in specific buffer for each protein (Table S4). After the endogenous peroxidase activity inactivation in 3% H_2_O_2_ solution for 10 min, slides were blocked with horse serum diluted in antibody dilution at 1:50 for 20 min and incubated overnight with primary antibody (Table S4). Then, the slides were incubated for 30 min with post-primary block, followed 30 min with polymer. The 3,3′-diaminobenzidine (Sigma-Aldrich, Germany) was used as a chromogen, and slides were counterstained with haematoxylin and mounted with Entellan (Merck-Millipore, Germany). A positive control was also included (Table S4).

Immunoexpression was evaluated semi-quantitatively by a pathologist and categorized according to extension (between 0–100%) and intensity (between 0–3). Only nuclear immunoreactivity was considered. The score for extension was score 0 (<1% of immunoreactive cells), score 1 (1–10% of immunoreactive cells), score 2 (10–50% of immunoreactive cells), and score 3 (>50% of immunoreactive cells). The score for intensity was score 0 (absent immunoexpression), score 1 (immunoexpression less intense than in normal tissue), score 2 (immunoexpression similar to normal tissue), and score 3 (higher immunoexpression than in normal tissue). The extension and intensity scores were combined, and a final score was defined as cut-off to define positive cases. Pictures were taken in a microscope Olympus BX41 with a digital camera Olympus U-TV0.63XC using CellA software.

### 2.17. Statistical Analysis

Statistical analysis was performed using the GraphPad Prim 6.0 software (GraphPad Software Inc., San Diego, CA, USA). Non-parametric Mann–Whitney *U* test was used to compare two groups. For comparisons between three or more groups, non-parametric Kruskal–Wallis test was used, followed by Mann–Whitney *U* test for pairwise comparisons and Bonferroni’s correction, when applicable. For all in vitro experiments, four independent replicates were performed. Differences in SIRT1 and NCAD immunoexpression between normal kidney, ccRCC, and pRCC tissues was assessed by chi-squared or Fisher’s exact test.

*p*-values were considered statistically significant when inferior to 0.05. Significance is shown vs. the respective control and depicted as follows: * *p* < 0.05, ** *p* < 0.01, *** *p* < 0.001, **** *p* < 0.0001, and ^ns^ *p* > 0.05 (non-significant).

## 3. Results

### 3.1. Lactate Decreased SIRT1 Expression, Increasing Cell Migration and Invasion in RCC

The effect of lactate was assessed in one primary and one metastatic clear cell RCC (ccRCC) (786-O and Caki-1, respectively) and papillary RCC (pRCC) (Caki-2 and ACHN, respectively) cell lines exposed to 20 mM lactate, which simulated the levels produced by glycolytic cells and released to the tumor microenvironment.

**Figure 1:**
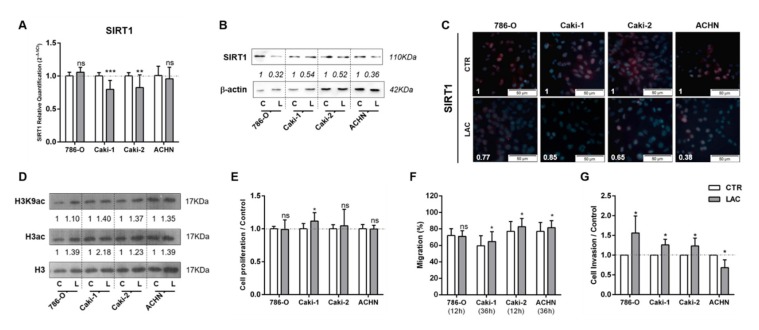
Lactate decreased sirtuin (SIRT)1’s expression and increased renal cell carcinoma (RCC) cell line aggressiveness. Characterization of SIRT1 expression in kidney tumor cell lines treated with 20 mM lactate by RT-qPCR (**A**), Western blot (**B**), and immunofluorescence (**C**). Characterization of global H3 acetylation and H3K9-specific mark in kidney tumor cell lines treated with 20 mM lactate by Western blot (**D**). Effect of 20 mM lactate treatment in kidney tumor cell lines at cell proliferation (5-bromo-2’-deoxyuridine (BrdU) assay) (**E**), cell migration (wound-healing assay), (**F**) and cell invasion (Matrigel Invasion Chambers) (**G**). Western blot and immunofluorescence quantification are represented as fold change of 20 mM lactate versus control condition; * *p* < 0.05, ** *p* < 0.01, *** *p* < 0.001, and ^ns^ *p* > 0.05 (non-significant).Abbreviations: C/CTR—control, L/LAC—20 mM lactate.

At the molecular level, lactate significantly decreased *SIRT1* expression levels in Caki-1 and Caki-2 lines (Figure 1A). The inhibitory effect of lactate on SIRT1 expression was also observed at the protein level for cells exposed to lactate in RCC cell lines tested (Figure 1B). Furthermore, a decrease in SIRT1 nuclear protein localization (Figure 1C) was also shown. Accordingly, lactate exposure increased global histone H3 and H3K9 acetylation levels for all cell lines (Figure 1D and Figure S1A). Moreover, without significant effect, a decrease in global sirtuin activity was observed, except for 786-O cells (Figure S2A).

However, with exception of Caki-1, lactate exposure did not significantly affect proliferation (Figure 1E). Conversely, lactate exposure increased migration capacity for most RCC cell lines (Figure 1F). Indeed, cell invasion was increased by 60% in 786-O cells exposed to lactate, and 25% in Caki-1 and Caki-2 cells (Figure 1G), whereas a 30% decrease was observed for ACHN cells exposed to lactate (Figure 1G).

### 3.2. Tumor Metabolism Modulated Epigenetic Landscape of Normal Adjacent Cells

**Figure 2:**
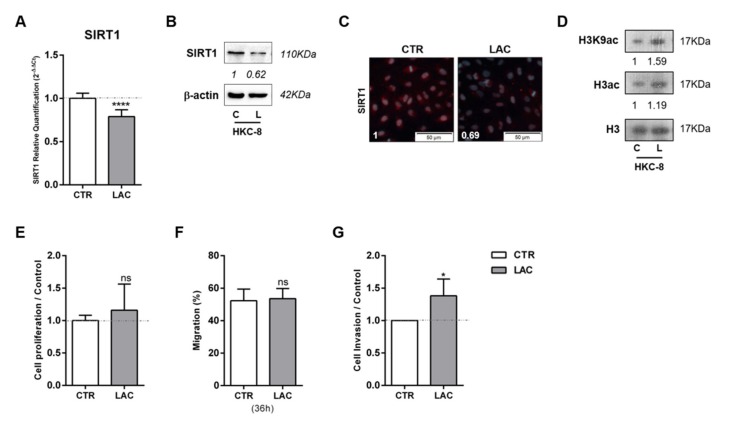
Lactate induced SIRT1 downregulation, contributing to normal kidney cell line pseudo-transformation. Characterization of SIRT1 expression in normal kidney cell line treated with 20 mM lactate by RT-qPCR (**A**), Western blot (**B**), and immunofluorescence (**C**). Characterization of global histone acetylation of histone H3 and specific histone marker H3K9 in normal kidney cell lines treated with 20 mM lactate by Western blot (**D**). Effect of 20 mM lactate treatment in cell proliferation (BrdU assay) (**E**), cell migration (wound-healing assay) (**F**), and cell invasion (Matrigel Invasion Chambers) (**G**). Western blot and immunofluorescence quantification are represented as fold change of 20 mM lactate versus control condition; * *p* < 0.05, **** *p* < 0.0001, and ^ns^ *p* > 0.05 (non-significant). Abbreviations: C/CTR—control, L/LAC—20 mM lactate.

In line with the results for cancer cell lines, HKC-8 normal kidney cell line exposed to 20 mM lactate displayed reduced *SIRT1* transcript (Figure 2A) and protein (Figure 2B,C) levels, as well as global sirtuin activity reduction (Figure S2B). Conversely, increased acetylated H3 and H3K9 levels were found (Figure 2D and Figure S1B). Despite no cell proliferation or migration changes being observed (Figure 2E,F, respectively), cell invasion was 38% higher in lactate-exposed HKC-8 cells compared to control cells (Figure 2G).

**Figure 3:**
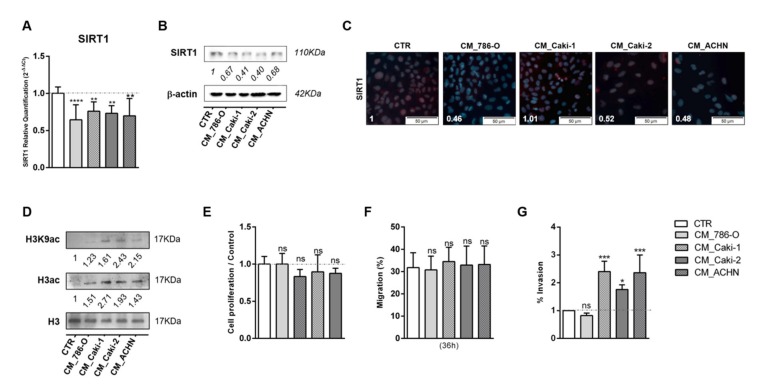
Conditioned medium (CM) modulated SIRT1, increasing histone acetylation and cell invasion of normal kidney cell line. Characterization of SIRT1 expression in normal kidney cell line treated with conditioned medium from tumor cells, by RT-qPCR (**A**), Western blot (**B**), and immunofluorescence (**C**). Characterization of global histone acetylation of histone H3 and specific histone marker H3K9 (**D**) in normal kidney cell line treated with conditioned medium from tumor cells, by Western blot. Effect of conditioned medium from tumor cells in normal kidney cell line at cell proliferation (BrdU assay) (**E**), cell migration (wound-healing assay) (**F**), and cell invasion (Matrigel Invasion Chambers) (**G**). Western blot and immunofluorescence quantification are represented as fold change of 20 mM lactate versus control condition; * *p* < 0.05, ** *p* < 0.01, *** *p* < 0.001, **** *p* < 0.0001 and ^ns^ *p* > 0.05 (non-significant). Abbreviations: CM—conditioned medium, CTR—control.

The effect of lactate produced and exported by cancer cells in epigenetic modulation of adjacent normal cells was also tested through exposure of HKC8 cells to CM of tumor cells collected after 72 h of culture. After 48 h of normal kidney cell line exposure to CM, *SIRT1* transcript and protein levels were significantly decreased in HKC-8 cell line for all conditions (Figure 3A–C). Moreover, in agreement with previous results, increased global histone 3 acetylation and H3K9 acetylation (Figure 3D and Figure S1C) were found. HKC-8 cells treated with CM did not affect cell proliferation rates (Figure 3E). In contrast, HKC-8 cell lines displayed a significant increase in cell invasion, but not migration, when exposed to some specific CM (Figure 3F,G). Thus, normal cell line exposed to tumor cells’ CM exhibited identical alterations to those induced by lactate treatment.

### 3.3. Treatment with SIRT1 Inhibitor, NAM, Mimicked the Effect of Lactate in Kidney Cells

**Figure 4:**
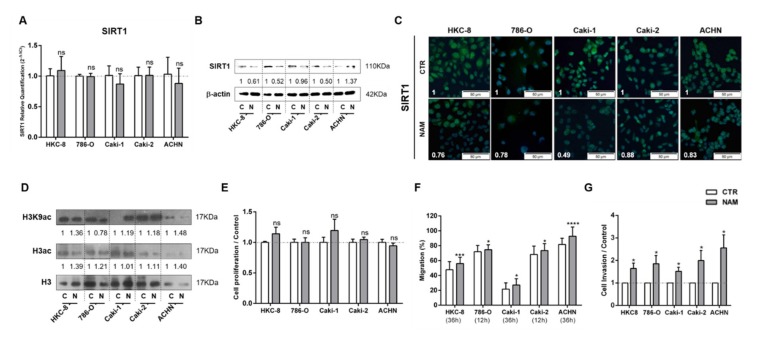
Nicotinamide (NAM) mimicked lactate effect in kidney cell lines. Characterization of SIRT1 expression by RT-qPCR (**A**), Western blot (**B**), and immunofluorescence (**C**). Characterization of global histone acetylation of histone H3 and specific histone marker H3K9 (**D**) by Western blot. Effect at cell proliferation by BrdU assay (**E**), cell migration by wound-healing assay (**F**), and cell invasion by Matrigel invasion chambers (**G**). Western blot and immunofluorescence quantification are represented as fold change of 200 µM nicotinamide versus control condition; * *p* < 0.05, *** *p* < 0.001, **** *p* < 0.0001 and ^ns^ *p* > 0.05 (non-significant) Abbreviations: C/CTR—control, N/NAM—200 µM nicotinamide.

The effect of SIRT1 inhibition in cell phenotype was also tested by treating cells with NAM. Globally, NAM treatment paralleled the effect of lactate in kidney cells. Although no evident effect was observed in *SIRT1* transcript levels (Figure 4A), it was associated with decreased SIRT1 protein levels (Figure 4B,C), and subsequently increased histone acetylation levels (Figure 4D and Figure S3). In addition, NAM promoted a decrease on global sirtuin activity in RCC, except for Caki2 cell line and normal kidney cells (Figure S2C). Notwithstanding, NAM did not induce significant changes in proliferation rates (Figure 4E). Moreover, a significant increase in cell migration (Figure 4F) and cell invasion (Figure 4G) was observed after NAM treatment in all tested RCC and normal kidney cell lines.

### 3.4. Lactate Promoted EMT through SIRT1-Dependent SMAD4 Axis

**Figure 5:**
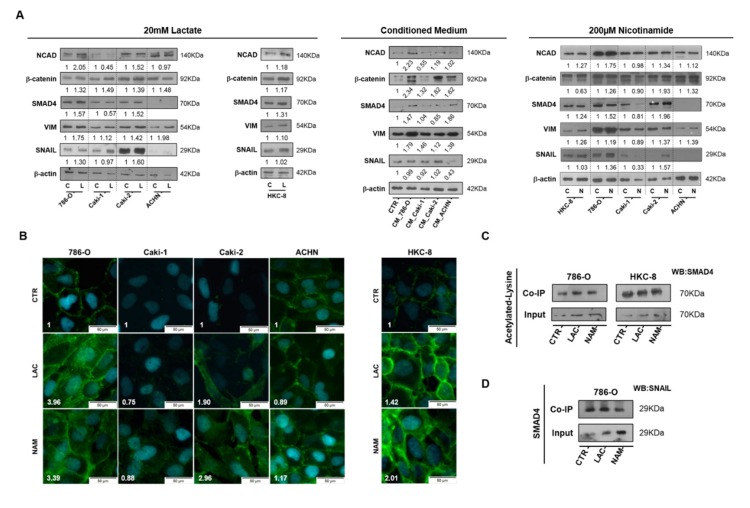
Lactate promoted epithelial–mesenchymal transition (EMT) phenotype through SIRT1-dependent SMAD4 axis in RCC cell lines. Characterization of EMT phenotype after 20 mM lactate, tumor cell-derived CM, and 200µM NAM treatment by Western blot (**A**). N-cadherin expression with 20 Mm lactate and 200µM NAM by immunofluorescence (**B**). Co-immunoprecipitation of acetylated lysine/SMAD4 (**C**) and SMAD4/SNAIL (**D**) in lactate and nicotinamide treatment conditions. Western blot and immunofluorescence quantification are represented as fold change of 20 mM lactate or 200µM nicotinamide versus control condition Abbreviations: C/CTR—control, CM—conditioned medium, L/LAC—20 mM lactate, N/NAM—200µM nicotinamide, NCAD—N-cadherin, VIM—vimentin.

In accordance with the observations for cell migration and cell invasion, both 20 mM lactate and 200µM NAM treatment increased N-cadherin (NCAD) expression in primary ccRCC (786-O), primary pRCC (Caki2), and normal HKC8 normal cell lines (Figure 5A,B). Additionally, increased expression of SMAD Family Member 4 (SMAD4) and vimentin were observed for RCC and HKC8 cell lines (Figure 5A). Enhanced Zinc finger protein SNAI1 (SNAIL) expression was only observed in RCC cell lines (Figure 5A). Furthermore, 786-O cell-derived CM increased N-cadherin, SMAD4, and vimentin expression in normal HKC8 cell line (Figure 5A). Moreover, increased β-catenin expression was observed in almost all considered conditions (Figure 5A). An enhancement in SMAD4 acetylation was observed for lactate and NAM studied conditions, particularly in 786-O cell line, through co-immunoprecipitation of acetylated lysine’s with SMAD4 protein (Figure 5C). However, no significant differences were observed for SMAD4 and SNAIL protein interaction with lactate and NAM treatment compared to control conditions (Figure 5D).

### 3.5. Lactate Transport Inhibition Increased SIRT1 Expression in RCC and Normal Kidney Cell Lines, Impacting Aggressiveness

**Figure 6:**
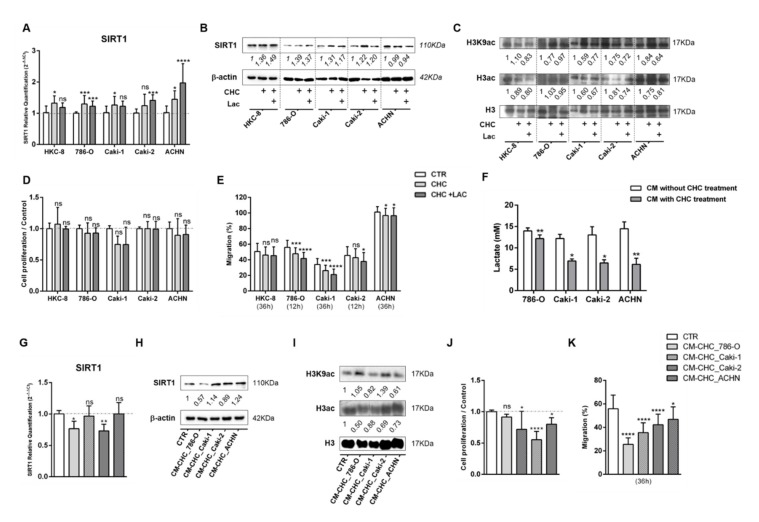
Alpha-cyano-4-hydroxycinnamate (CHC) reverted the lactate effect in normal kidney cell lines. Characterization of SIRT1 expression by RT-qPCR (**A**,**G**) and Western blot (**B**,**H**). Characterization of global histone acetylation of histone H3 and specific histone marker H3K9 (**C**,**I**) by Western blot. Effect at cell proliferation (**D**,**J**) by BrdU assay and cell migration (**E**,**K**) by wound-healing assay. Measurement of lactate levels after CHC treatment in RCC cell lines (**F**); * *p* < 0.05, ** *p* < 0.01, *** *p* < 0.001, , **** *p* < 0.0001 and ^ns^ *p* > 0.05 (non-significant) Abbreviations: CTR—control, CHC—alpha-cyano-4-hydroxycinnamate, CM—conditioned medium, LAC—20 mM lactate.

To revert lactate effects, kidney cell lines were treated with MCT inhibitor CHC for 48 h, and the IC_50_ values were calculated (Figure S4). Indeed, CHC associated with increased SIRT1 expression (Figure 6A,B and Figure S5), whereas histone acetylation levels were decreased (Figure 6C and Figure S5). Furthermore, although not statistically significant, CHC exposure was associated with diminished cell growth in 786-O, Caki2, and ACHN cells (Figure 6D). Except for the HKC-8 cell line, CHC treatment significantly decreased cell migration capacity (Figure 6E).

To further confirm the fact that the effect of RCC cells’ CM observed upon normal cells was due to the presence of lactate, normal cells were treated with CM from RCC cells previously exposed to CHC (CM-CHC). Indeed, lactate quantity in CM-CHC was significantly reduced compared with CM (Figure 6F). Accordingly, a slight increase in transcript and protein SIRT1 levels were observed (Figure 6G,H and Figure S6A), followed by a small decrease in H3K9 and H3 acetylation levels for Caki1 and ACHN cells (Figure 6I and Figure S6B). Importantly, decreased cell proliferation (Figure 6J) and cell migration (Figure 6K) were observed in almost all studied CHC-CM conditions.

**Figure 7:**
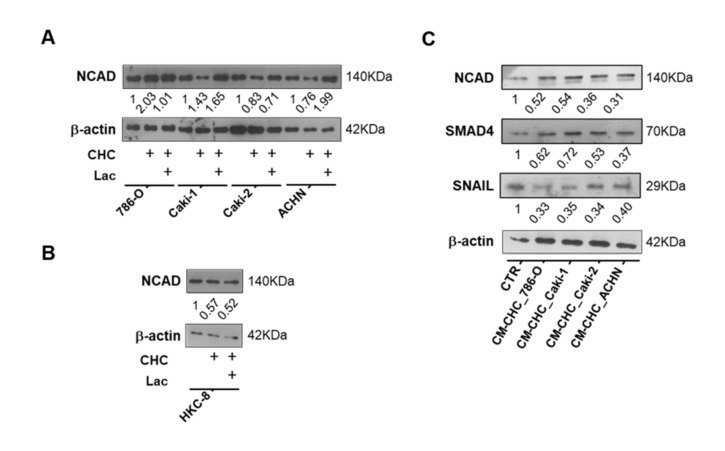
Monocarboxylate transporter (MCT) inhibition reverted the lactate-derived EMT effect in RCC and normal kidney cell lines. Characterization of N-cadherin expression after CHC treatment in (**A**) RCC cell lines and (**B**) normal kidney cell line, by Western blot. N-cadherin, SMAD4, and SNAIL expression in HKC8 cell lines after CHC tumor cell line-derived CM treatment (**C**). Abbreviations: CTR—control, CHC—alpha-cyano-4-hydroxycinnamate, CM—conditioned medium, LAC—20 mM lactate, NCAD—N-cadherin.

Moreover, a decrease in N-cadherin expression was observed in Caki2 and HKC8 cell lines exposed to CHC and CHC+LAC conditions (Figure 7A,B). Similarly, N-cadherin expression was decreased in HKC8 exposed to CM-CHC (Figure 7C), followed by reduced SMAD4 and SNAIL expression (Figure 7C).

### 3.6. In Vivo Effect of Lactate, NAM, and CHC on RCC Cells

**Figure 8:**
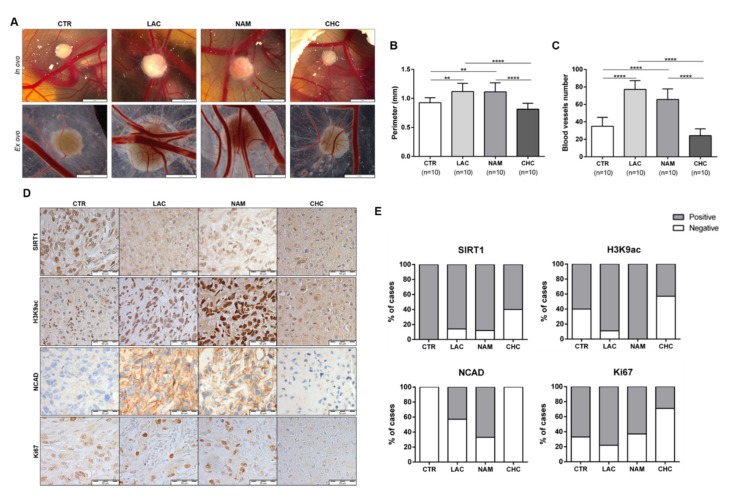
In vivo effect of lactate, NAM, and CHC upon RCC microtumors. Representative *in ovo* and *ex ovo* pictures of chick chorioallantoic membrane (CAM) assay at final timepoint (**A**). Quantification of tumor growth (**B**) and total of blood vessels (**C**) in CAM tumors. Representative pictures (**D**) and graphics (**E**) of immunoexpression in CAM-developed tumors according to exposure to chemicals; ** *p* < 0.01, *** *p* < 0.001, **** *p* < 0.0001 and ^ns^ *p* > 0.05 (non-significant) Abbreviations: CTR—control, CHC—alpha-cyano-4-hydroxycinnamate, LAC—20 mM lactate, NAM—200µM nicotinamide, NCAD—N-cadherin.

Using in vivo CAM assay, lactate and NAM treatments significantly increased tumor size and blood vessel recruitment in 786-O cell line (Figure 8A–C). No significant differences were observed between cells exposed to lactate and NAM, suggesting that lactate action upon tumor cells is partially accomplished through SIRT regulation. In contrast, CHC treatment reversed these effects in CAM-developed tumors (Figure 8A–C).

Lactate exposure associated with decreased expression of SIRT1 in CAM-developed tumors (Figure 8D,E and Table S5), which was paralleled by increased H3K9 acetylation levels (Figure 8D,E and Table S5). NAM treatment was associated with decreased SIRT1 levels, although H3K9ac was positive for all the CAM-developed tumors (Figure 8D,E and Table S5). Importantly, NCAD expression was only observed in lactate and NAM groups (Figure 8D,E and Table S5), implicating SIRT1 in EMT modulation and aggressiveness.

Moreover, CAM-developed tumors treated with CHC revealed reduced SIRT1 expression (Figure 8D,E and Table S5), but also lower H3K9ac expression (Figure 8D,E and Table S5). In the same line of BrdU assay results, in in vitro tests, lactate and NAM treatment did not change Ki67 expression, whereas decreased Ki67 positivity was observed in CAM-developed tumors exposed to CHC (Figure 8D,E and Table S5). Hence, in vivo observations fully supported the in vitro findings.

### 3.7. SIRT1 and N-Cadherin Protein Levels in Primary Tumors

**Figure 9:**
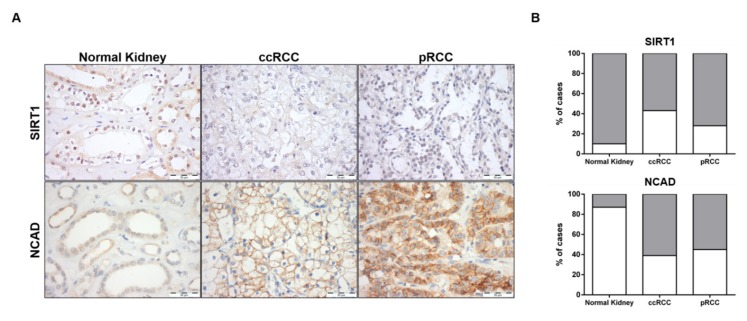
Characterization of SIRT1 and N-cadherin in kidney tissues. Expression of SIRT1 and NCAD in normal kidney and renal cell carcinoma tissues (clear cell RCC (ccRCC) and papillary RCC (pRCC) cases) by immunohistochemistry (**A**). Graphical representation of immunoexpression positive and negative cases (**B**). Abbreviations: ccRCC—clear cell renal cell carcinoma, pRCC—papillary renal cell carcinoma.

SIRT1 and N-cadherin characterization was also performed in normal kidney and RCC tissues. The proportion of cases defined as “positive” and immunoexpression intensity scores were compared between normal kidney and RCC tissues for SIRT1 and NCAD. Only nuclear SIRT1 expression and plasma membrane NCAD expression were considered (Figure 9A).

As expected, a significant increase in NCAD was found in ccRCC and pRCC (60.5% (23/38), *p* = 0.0001 and 55.3% (21/38), *p* = 0.0004, respectively) compared to normal kidney tissues (13.3% (4/30)) (Figure 9B and Table S6). Regarding SIRT1, decreased expression levels was depicted by RCC compared to normal tissues, with 90.0% (27/30) of normal tissues displaying SIRT1 positivity, compared to 57.5% (23/40), *p* = 0.0033, and 71.8% (28/39), *p* = 0.0764, in ccRCC and pRCC, respectively (Figure 9B and Table S6). Indeed, a significant inverse association was observed between NCAD and SIRT1 protein levels (*p* = 0.0266) in RCC tissues (Table S7).

## 4. Discussion

Renal cell carcinomas (RCCs) are the most lethal of the common urological cancers, with metastasis being the foremost cause of cancer-related mortality. Despite improvements in adjuvant therapies, the impact on survival of patients with locally advanced and metastatic disease has been feeble [30]. Hence, in-depth understanding of the mechanisms underlying tumor progression, including metastization, is imperative to develop new therapeutic strategies for advanced stage RCC. Highly glycolytic tumors, including RCCs, are characterized by increased production of lactate [7], a major oncometabolite resulting from tumor metabolism rewiring, which has a relevant impact in cancer aggressiveness and therapy resistance [8,31]. Intratumoral lactate levels seem to be prognostic in several cancers. In fact, it has been demonstrated that high lactate levels are associated with higher incidence of distant metastasis in different cancer types [11], although the mechanism is not completely understood.

An interplay between tumor metabolites and epigenetic landscape has recently emerged, indicating that metabolism is closely associated with altered epigenetic landscape in cancer cells [32,33]. Indeed, lactate was suggested as modulating epigenetic mechanisms, namely, reduction of HDAC class I and II activity in colorectal and cervical cancer models [17,18]. Because class III/sirtuins’ activity is NAD^+^-dependent and considering the high glycolytic rate of RCC cells and high lactate levels in the tumor microenvironment, a decrease in NAD^+^/NADH is expected, entailing SIRT activity inhibition [7,34]. Thus, a role for lactate in SIRT inhibition was hypothesized. Herein, we evaluated the effects of lactate in SIRT1 and consequent alterations in RCC phenotype. Remarkably, we found that lactate decreases SIRT1 expression and activity in RCC cell lines. Because SIRT1 targets H3K9 [35], we also assessed the global histone acetylation levels and the specific histone marks in cell lines exposed to lactate. As expected, lactate demonstrated a reversible effect on histone deacetylation levels and induced histone hyperacetylation in in vitro and in vivo conditions, putatively as a consequence of SIRT1 inhibition. We also observed a more striking increase in the H3K9ac mark. These findings suggest that hyperacetylation of histone H3 and H3K9 might be involved in gene transcription regulation. Interestingly, histone acetylation was shown to be involved in transcriptional regulation of EMT-related genes and associated pathways [36,37,38], and lactate increases H3K9ac mark, as well as global histone acetylation [17].

Currently it is acknowledged that lactate is not just a waste metabolite but an important oncometabolite in the tumor microenvironment that is involved in tumor aggressiveness and metastasis [9]. However, the underlying mechanism is not well understood, and a metabolic–epigenetic relationship has been proposed. In cervical cancer, lactate inhibits HDAC activity and increases H3 and H4 acetylation levels, modulating cellular DNA damage repair processes and affecting therapy response [18]. This suggests HDAC regulation by lactate and consequent involvement in gene transcription control, which may contribute to tumor progression and therapy resistance. In our study, we demonstrated that lactate modulates SIRTs, decreasing SIRT1 expression. Additionally, although lactate did not affect cell growth/proliferation, increased cell migration and cell invasion was observed. These findings are supported by previous studies that associated increased tumor aggressiveness with SIRT1 downregulation [39,40].

Sirtuins have been implicated in different cellular processes, being key epigenetic modulators of EMT activation and maintenance [23,24]. Interestingly, SIRT1 has been shown to play a dual role in tumorigenesis, acting either as an oncogene or tumor suppressor gene according to the tumor context [23,27]. Specifically, in lung and ovarian cancers, SIRT1 was shown to repress EMT via cell migration inhibition [41,42]. Furthermore, SIRT1 upregulation, through resveratrol treatment, inhibited EMT in renal injury and fibrosis [43]. In the same vein, we found that SIRT1 downregulation, due to lactate exposure or NAM treatment, increased N-cadherin and vimentin expression in RCC cell lines. Moreover, high SMAD4 acetylation levels were observed after lactate and NAM treatment, indicating that decreased SIRT1 deacetylation activity on SMAD4 favors migration and invasion in RCC cells. Indeed, SIRT1 was reported to suppress EMT and metastization by deacetylating SMAD4 [39,44]. Additionally, EMT inhibition by SIRT1 was reported in kidney tubular epithelial cells after Transforming growth factor- beta (TGF-β) treatment [39]. The effect of SIRT1 in EMT phenotype though SMAD4 deacetylation has also been reported in oral squamous cell carcinoma [40]. Conversely, SIRT1 loss of expression promoted SMAD4 hyperacetylation; increased MMP7 levels; and, consequently, caused reduction of E-cadherin expression, and SIRT1 knockdown (KD) prevented β-catenin degradation by promoting its translocation to the nucleus (a mesenchymal feature) [39]. Remarkably, in our hands, lactate and NAM treatment also increased β-catenin protein levels, sustaining a lactate-mediated SIRT1 downregulation in RCC cells, promoting an EMT phenotype.

The effect of lactate was further confirmed by the results observed with SIRT and MCT inhibitors (NAM and CHC, respectively). NAM is a noncompetitive inhibitor that blocks SIRTs deacetylation activity [45], disclosing anti-neoplastic effect in leukemia, oral squamous cell carcinoma, and prostate cancer by decreasing cell viability and proliferation, while inducing apoptosis [46,47]. Contrarily, in our study, SIRT1 inhibition by NAM increased malignant cell aggressiveness, promoting tumor progression. Nonetheless, CHC, a classical MCT inhibitor with more affinity for MCT1 [48], led to enhanced SIRT1 expression, decreased histone acetylation, and N-cadherin expression, associating with reduced cell migration in cancer and normal kidney cell lines. By preventing lactate uptake, CHC was able to revert SIRT1 inhibition and attenuate the malignant phenotype. Because CHC also inhibits lactate efflux with consequent reduction of glycolytic metabolism [49], NAD^+^ availability is augmented [50] allowing for SIRT1 reactivation. Indeed, several in vitro and in vivo studies highlighted the role of MCT inhibitors in cancer [51]. Remarkably, CHC attenuating impact on cancer cell aggressiveness was previously demonstrated in glioma and breast cancer cells [48,51].

Lactate produced and exported by tumor cells can be used by adjacent tumor cells, as well as other cells in the tumor microenvironment, including endothelial cells and stromal cancer-associated fibroblasts, reprogramming their functions and contributing to tumor progression [52,53]. Consequently, we hypothesized that lactate might also modulate the same epigenetic mechanisms in adjacent normal cells. We found that exposure of normal kidney cell lines to lactate and to CM of tumor cells entails cellular alterations similar to those observed in tumor cells. In fact, lactate uptake caused a decrease in SIRT1 expression, contributing to global hyperacetylation and, consequently, increased cell migration and invasion, as well as EMT. Nonetheless, treatment with CM entailed decreased cell growth and proliferation rates, probably due to acidic pH and low concentration of nutrients available in the medium. Remarkably, although lactate effect in CM was not totally abrogated by CHC, a partial effect was observed on SIRT1 levels and cell features. Thus, excess lactate in the tumor microenvironment seems to modulate epigenetic mechanisms of adjacent normal cells and, eventually, contribute to the acquisition of some malignant features in those cells, a phenomenon that might be referred to as “pseudo-transformation”, which might cooperate with neoplastic transformation and progression.

Globally, in vivo assays confirmed in vitro results. Indeed, lactate treatment led to more aggressive and angiogenic tumors, with larger tumor perimeters, as previously shown in glioma [51]. Moreover, decreased SIRT1 and increased H3K9 acetylation, along with increased NCAD expression, was also observed in CAM-developed tumors. NAM treatment also mimicked lactate’s effect in those tumors. Furthermore, CHC treatment only display a very mild effect in CAM-developed tumor growth, as well as in SIRT1 and H3K9 acetylation levels. Moreover, this treatment is associated with low Ki67 positivity, as previously reported [48], and absence of N-cadherin expression, supporting decreased CAM-developed tumor growth.

In primary RCC, SIRT1 expression was decreased compared with normal kidney tissues, particularly for ccRCC. Our results are in accordance with the only report available thus far [54]. Thus, our results further support a tumor-suppressive role of SIRT1 in RCC. In parallel, we observed an inverse association between SIRT1 with N-cadherin expression, suggesting a link between SIRT1 expression and invasive phenotype, and consequently metastization in RCC.

Globally, our data suggest a new mechanism—SIRT1-mediated—by which lactate contributes to RCC aggressiveness and pseudo-transformation of adjacent normal cells. Thus, we postulate that lactate produced by glycolytic tumor cells is exported across the cell membrane to the tumor microenvironment by MCTs and, according with tumor metabolic symbiosis theory, it might be used by adjacent tumor and normal cells through an NAD^+^-dependent pathway, leading to a decreased NAD^+^/NADH ratio, resulting in SIRT1 inhibition. This, in turn, would increase histone H3 hyperacetylation with consequent augment of tumor cell aggressiveness. Finally, considering our findings, especially the role of lactate in tumor microenvironment modulation, new therapeutic strategies for advanced RCC might be envisaged. Indeed, the use of MCTs inhibitors [31] or other drugs that restore SIRT1 activity (e.g., resveratrol [55]) should be explored in order to complement current therapies, improving RCC patient outcome.

## 5. Conclusions

We have shown that lactate contributes to increased RCC aggressiveness, likely through SIRT1 activity reduction. Furthermore, increased lactate levels in the tumor microenvironment also impact on normal cells, modulating their phenotype and bringing it closer to that of malignant cells (pseudo-transformation). Importantly, tumor metabolism was unveiled as a promising therapeutic target in RCC, deserving further exploration.

## Supplementary Materials

The following are available online at https://www.mdpi.com/2073-4409/9/4/1053/s1, Table S1: Clinical pathologic characterization of epithelial human kidney cell lines. Table S2: Primer used in RT-qPCR. Table S3: Primary antibodies used in Western blot (WB) and Immunofluorescence (IF). Table S4: Primary antibodies used in immunohistochemical (IHC) analysis. Table S5: Immunoexpression in CAM tissues. Table S6: SIRT1 and NCAD immunoexpression in normal kidney and RCC tissues. Table S7: Associations between NCAD and SIRT1 immunoexpression in RCC tissues. Figure S1: Lactate increase histone acetylation in RCC cell lines, normal kidney cell lines, and normal kidney cell lines treated with conditioned medium. Figure S2: Characterization of global SIRT activity in RCC and normal kidney cell lines treated with lactate and nicotinamide. Figure S3: Characterization of histone acetylation in kidney cell lines treated with 200 µM NAM. Figure S4: Growth inhibition by CHC. Figure S5: Characterization of SIRT1 and histone acetylation in kidney cell lines treated with CHC. Figure S6: Characterization of SIRT1 and histone acetylation in kidney cell lines treated with CM-CHC.
