# Supplementary material for: Lactate Increases Renal Cell Carcinoma Aggressiveness through Sirtuin 1-Dependent Epithelial Mesenchymal Transition Axis Regulation
Source: Cells. 2020 Apr 23;9(4):1053. doi: 10.3390/cells9041053 (PMC7226526; doi:10.3390/cells9041053)
Supplement: Supplementary file 1 [file cells-09-01053-s001.pdf]

Miranda-Gonçalves et al.

Supplementary Tables

Table S1. Clinical pathologic characterization of epithelial human kidney cell lines

Kidney cell lines	
**HKC-8**	Human renal proximal tubule epithelial cells
**786-O**	Primary clear cell renal cell carcinoma
**Caki-1**	Metastatic clear cell renal cell carcinoma (metastatic site: skin)
**Caki-2**	Primary papillary renal cell carcinoma
**ACHN**	Metastatic papillary renal cell carcinoma (metastatic site: pleural effusion)

Table S2. Primer used in RT-qPCR

Gene	Forward sequence (5′-3′)	Reverse sequence (5′-3′)	T annealing
SIRT1	GCGGGAATCCAAAGGATAAT	GCACCTAGGACATCGAGGAA	60ºC
β-GUS	CTCATTTGGAATTTTGCCGATT	CCGAGTGAAGATCCCCTTTTTA	60ºC

Table S3. Primary antibodies used in WB and IF

Primary antibody	Company	Western-Blot dilution	IF dilution	Second antibody specie
**H3**	Abcam, ab1791	1:5000, 5% BSA in TBS/T	n.a.	Anti-rabbit
**H3ac**	Millipore, 06-599	1:5000, 5% BSA in TBS/T	1:250	Anti-rabbit
**H3K9ac**	Millipore, SC20058	1:500, 5% BSA in TBS/T	1:150	Anti-rabbit
**N-cadherin**	Cell Signaling Technology, 1311	1.1000, 5% BSA in TBS/T	1.100	Anti-rabbit
**SIRT1**	Abcam, ab32441	1:1000, 5% milk in TBS/T	1:250	Anti-rabbit
**SMAD4**	Cell Signaling Technology, 38454	1:1000, 5% milk in TBS/T	n.a.	Anti-rabbit
**SNAIL**	Cell Signaling Technology, 3879	1:1000, 5% milk in TBS/T	n.a	Anti-rabbit
**Vimentin**	Leica, VIM-V9-LCE	1:1000, 5% milk in TBS/T	n.a	Anti-mouse
**β-Actin**	Sigma-Aldrich, A1978	1:10,000, 5% milk in TBS/T	n.a.	Anti-mouse
**β-Catenin**	Cell Signaling Technology, 8480	1:1000, 5% milk in TBS/T	n.a.	Anti-rabbit

IF – Immunofluorescence; n.a. – not applicable

Table S4. Primary antibodies used in IHC

Primary antibody	Company	Positive control	Antigen retrieval	Primary antibody dilution	Detection system
**H3K9ac**	Millipore, SC20058	Colon	Citrate buffer (10mM, pH=6), microwave	1:750 overnight RT	Novolink™ Max Polymer Detection System
**Ki67**	M7240, DAKO	Breast cancer	Citrate buffer (10mM, pH=6), water bath	1:200 1h RT	UltraVision Detection System
**N-cadherin**	Cell Signaling Technology, 1311	Testis	EDTA buffer (1mM, pH=8), microwave	1:500 overnight RT	Novolink™ Max Polymer Detection System
**SIRT1**	Abcam, ab32441	Colon	EDTA buffer (1mM, pH=8), microwave	1:350 overnight RT	Novolink™ Max Polymer Detection System

RT – Room temperature

Table S5. Immunoexpression in CAM tissues

		CTR	LAC	NAM	CHC
	n	7	7	8	5
**SIRT1**	Negative (%)	0 (0)	1 (14.3)	1 (12.5)	2 (40.0)
	Positive (%)	7 (100)	6 (85.7)	7 (87.5)	3 (60.0)
	n	6	8	9	5
**NCAD**	Negative (%)	6 (100)	5 (57.1)	3 (33.0)	5 (100)
	Positive (%)	0 (0)	3 (42.9)	6 (67.0)	0 (0)
	n	5	9	9	7
**H3K9ac**	Negative (%)	2 (40.0)	1 (11.1)	0 (0)	4 (57.1)
	Positive (%)	3 (60.0)	8 (88.9)	9 (100)	3 (42.9)
	n	6	9	8	7
**Ki67**	Negative (%)	2 (33.3)	4 (44.4)	3 (37.5)	5 (71.4)
	Positive (%)	4 (66.7)	5 (55.6)	5 (62.5)	2 (28.6)

Table S6. SIRT1 and NCAD immunoexpression in normal kidney and RCC tissues

		Normal Kidney	ccRCC	pRCC
	n	30	40	39
**SIRT1**	Negative (%)	3 (10.0)	17 (42.5)	11 (28.2)
	Positive (%)	27 (90.0)	23 (57.5)	28 (71.8)
	p value	n.a.	0.0033	0.0764
	n	30	38	38
**NCAD**	Negative (%)	26 (86.7)	15 (39.5)	17 (44.7)
	Positive (%)	4 (13.3)	23 (60.5)	21 (55.3)
	p value	n.a.	0.0001	0.0004

ccRCC – Clear cell Renal Cell Carcinoma; pRCC – Papillary Renal Cell Carcinoma

Table S7. Associations between NCAD and SIRT1 in RCC tissues

		NCAD		
		Negative	Positive	*p value*
	n	75		
**SIRT1**	Negative (%)	6 (8.0)	19 (25.3)	0.0266
	Positive (%)	26 (34.7)	24 (32.0)	

Supplementary figures

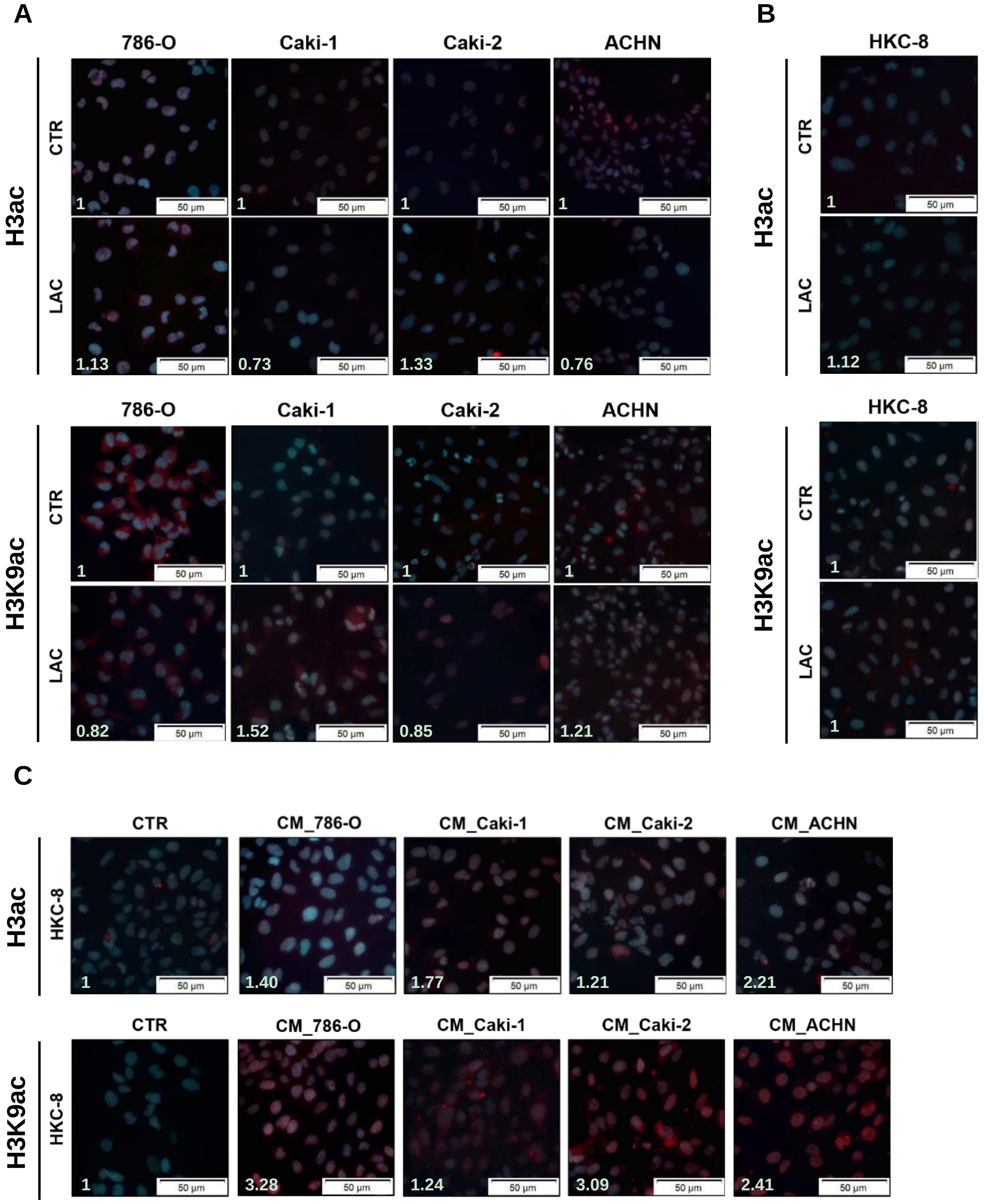

Figure S1: Characterization of total global H3 acetylation and specific histone markers H3K9 in RCC cell lines **(A)** and normal kidney cell lines **(B)** treated with 20mM lactate, and in normal kidney cell lines treated with conditioned medium from tumor cells **(C)**. Fluorescence intensity ratio (white) for different studied conditions was normalized to control condition. Abbreviations: CTR – Control, Lac – 20mM lactate, CM – Conditioned medium.

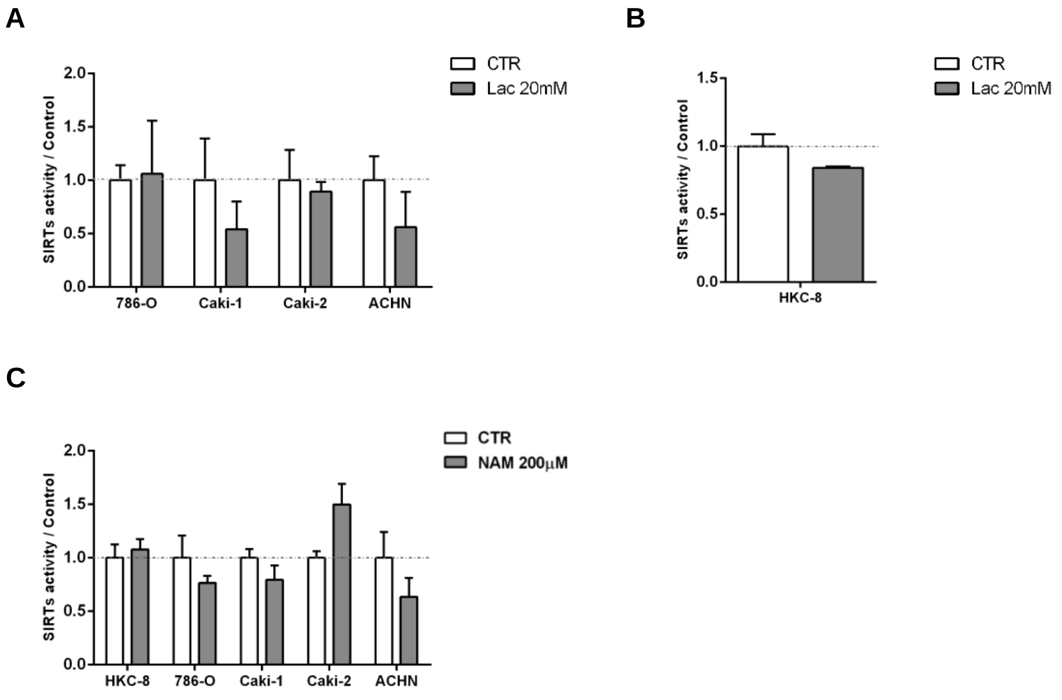

Figure S2: Characterization of sirtuin activity in RCC (A) and normal kidney cells (B) treated with 20mM lactate, as well as 200μM NAM (C). Abbreviations: CTR – Control, Lac – 20mM lactate, NAM – 200μM nicotinamide.

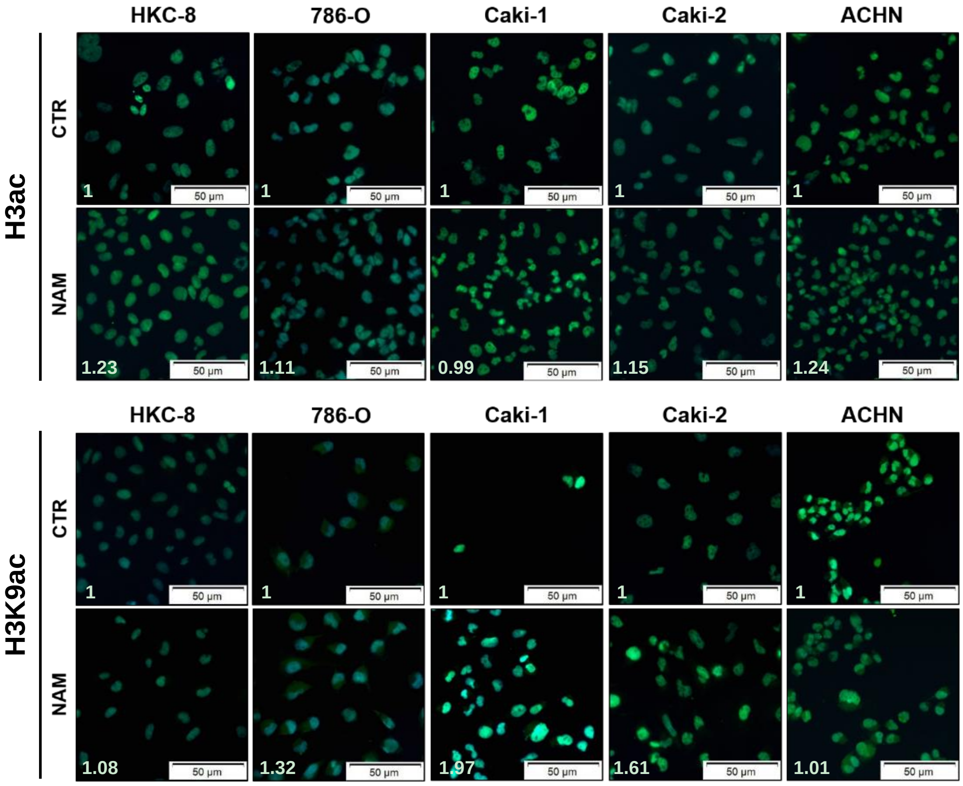

Figure S3. Characterization of global H3 acetylation and specific histone markers H3K9 protein expression in kidney cell lines treated with 200µM NAM. Fluorescence intensity ratio (white) for NAM condition was normalized to control condition. Abbreviations: CTR – Control, NAM – 200µM nicotinamide.

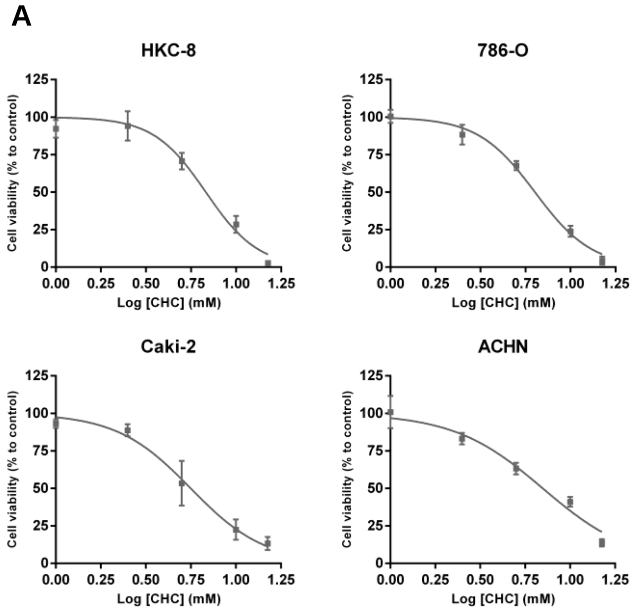

B

	IC_{50} (mM) ± SD
HKC-8	6.7± 0.5
786-O	6.3 ± 0.1
Caki-1	5.1 ± 0.2
Caki-2	6.6 ± 1.7
ACHN	6.9 ± 0.8

Figure S4. Growth inhibition by CHC. Cell viability curves after 48h of CHC treatment (1, 2.5, 5, 10 and 15mM) **(A)** and IC_{50} values (mean±SD) **(B)** for kidney cell lines.

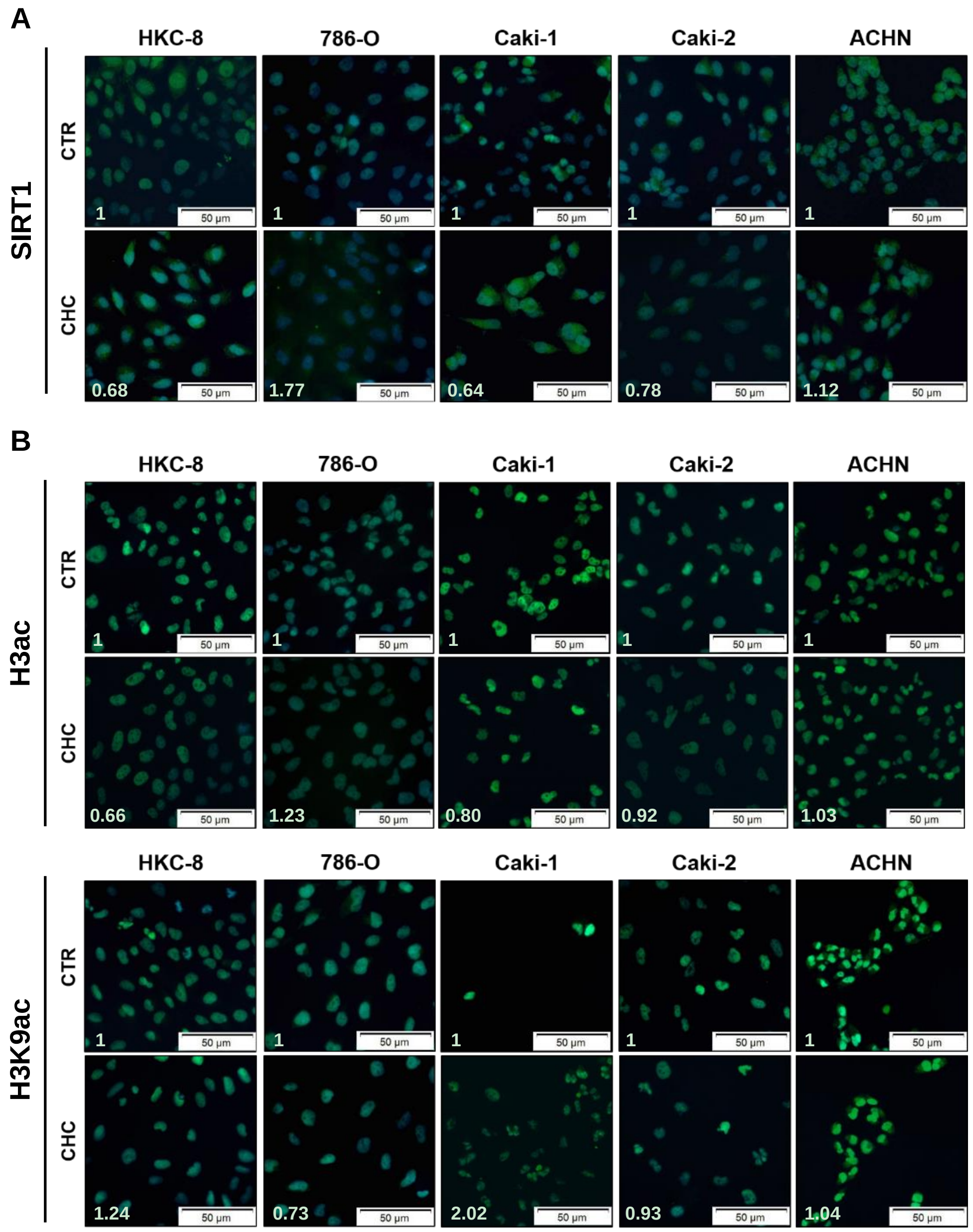

Figure S5. Characterization of SIRT1 **(A)**, and global H3 acetylation and specific histone markers H3K9 **(B)** protein expression in kidney cell lines treated with CHC. Fluorescence intensity ratio (white) for CHC condition was normalized to control condition. Abbreviations: CTR – Control, CHC – Alpha-cyano-4-hydroxycinnamate.

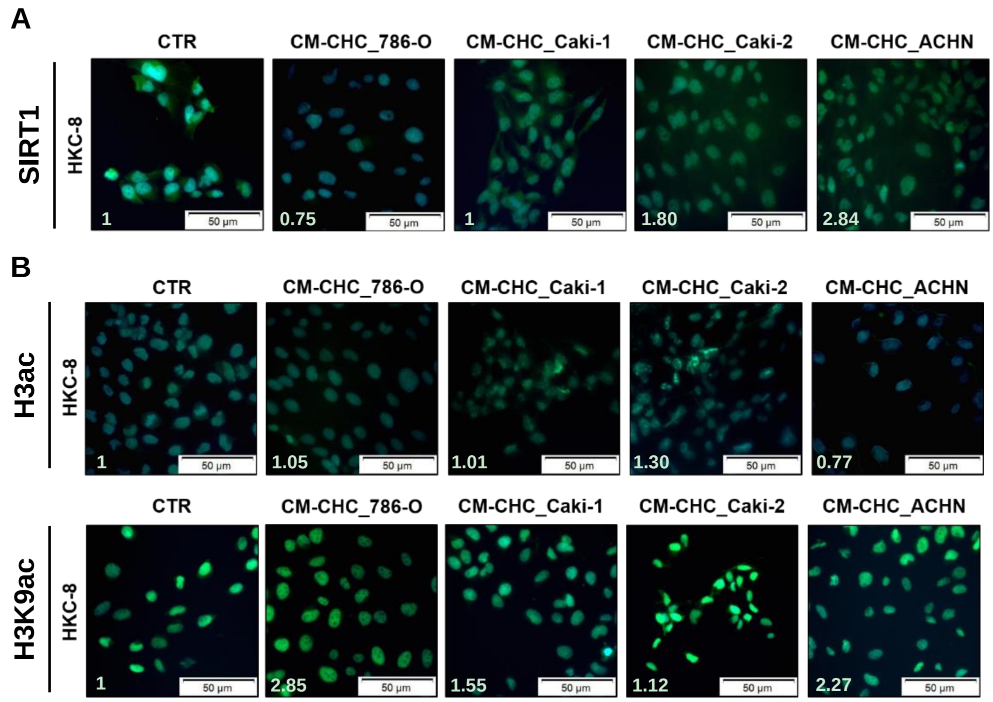

Figure S6. Characterization of SIRT1 **(A)**, and global H3 acetylation and specific histone markers H3K9 **(B)** protein expression in kidney cell lines treated with CM-CHC. Fluorescence intensity ratio (white) for CM-CHC condition was normalized to control condition. Abbreviations: CTR – Control, CM-CHC – CM from tumor cells previously treated with alpha-cyano-4-hydroxycinnamate (CHC).
